# Supplementary material for: Modeling glioblastoma heterogeneity as a dynamic network of cell states
Source: Mol Syst Biol. 2021 Sep 16;17(9):e10105. doi: 10.15252/msb.202010105 (PMC8444284; doi:10.15252/msb.202010105)
Supplement: Supplementary file 5 — Source Data for Figure 3 [file MSB-17-e10105-s001.zip › Figure3A_sourcedata/GSEA_3065/hallmarks_state6.GseaPreranked.1623416308702/HALLMARK_ESTROGEN_RESPONSE_EARLY.html]

Details for gene set HALLMARK\_ESTROGEN\_RESPONSE\_EARLY[GSEA]

|  || Dataset | state6 |
| Phenotype | NoPhenotypeAvailable |
| Upregulated in class | na\_neg |
| GeneSet | HALLMARK\_ESTROGEN\_RESPONSE\_EARLY |
| Enrichment Score (ES) | -0.30573994 |
| Normalized Enrichment Score (NES) | -1.2247726 |
| Nominal p-value | 0.09966216 |
| FDR q-value | 0.23354413 |
| FWER p-Value | 0.985 |
Table: GSEA Results Summary

  

Fig 1: Enrichment plot: HALLMARK\_ESTROGEN\_RESPONSE\_EARLY      
 Profile of the Running ES Score & Positions of GeneSet Members on the Rank Ordered List

  

| PROBE | GENE SYMBOL | GENE\_TITLE | RANK IN GENE LIST | RANK METRIC SCORE | RUNNING ES | CORE ENRICHMENT || 1 | RAB31 |  |  | 23 | 0.284 | 0.0404 | No |
| 2 | KLF10 |  |  | 234 | 0.166 | 0.0414 | No |
| 3 | RHOBTB3 |  |  | 249 | 0.162 | 0.0644 | No |
| 4 | PPIF |  |  | 465 | 0.127 | 0.0589 | No |
| 5 | SVIL |  |  | 471 | 0.126 | 0.0775 | No |
| 6 | BAG1 |  |  | 557 | 0.117 | 0.0856 | No |
| 7 | KDM4B |  |  | 629 | 0.112 | 0.0944 | No |
| 8 | SLC1A4 |  |  | 741 | 0.103 | 0.0972 | No |
| 9 | TOB1 |  |  | 746 | 0.102 | 0.1122 | No |
| 10 | TUBB2B |  |  | 795 | 0.099 | 0.1217 | No |
| 11 | BLVRB |  |  | 928 | 0.090 | 0.1201 | No |
| 12 | RPS6KA2 |  |  | 1117 | 0.080 | 0.1106 | No |
| 13 | PMAIP1 |  |  | 1232 | 0.074 | 0.1088 | No |
| 14 | CCND1 |  |  | 1317 | 0.071 | 0.1099 | No |
| 15 | FAM102A |  |  | 1404 | 0.067 | 0.1102 | No |
| 16 | ABAT |  |  | 1484 | 0.065 | 0.1109 | No |
| 17 | TFAP2C |  |  | 1552 | 0.062 | 0.1126 | No |
| 18 | WWC1 |  |  | 1563 | 0.062 | 0.1209 | No |
| 19 | ADCY9 |  |  | 1597 | 0.061 | 0.1263 | No |
| 20 | ITPK1 |  |  | 1637 | 0.059 | 0.1307 | No |
| 21 | NBL1 |  |  | 1808 | 0.053 | 0.1193 | No |
| 22 | BHLHE40 |  |  | 1880 | 0.051 | 0.1189 | No |
| 23 | ARL3 |  |  | 2076 | 0.046 | 0.1034 | No |
| 24 | MYC |  |  | 2081 | 0.045 | 0.1098 | No |
| 25 | SH3BP5 |  |  | 2154 | 0.043 | 0.1081 | No |
| 26 | UNC119 |  |  | 2488 | 0.034 | 0.0749 | No |
| 27 | NCOR2 |  |  | 2497 | 0.033 | 0.0790 | No |
| 28 | ELF1 |  |  | 2696 | 0.029 | 0.0607 | No |
| 29 | CELSR2 |  |  | 2741 | 0.028 | 0.0599 | No |
| 30 | SCARB1 |  |  | 2923 | 0.024 | 0.0426 | No |
| 31 | DYNLT3 |  |  | 3160 | 0.019 | 0.0183 | No |
| 32 | MAPT |  |  | 3232 | 0.017 | 0.0127 | No |
| 33 | TSKU |  |  | 3253 | 0.016 | 0.0129 | No |
| 34 | SEC14L2 |  |  | 3269 | 0.016 | 0.0136 | No |
| 35 | NAV2 |  |  | 3288 | 0.016 | 0.0139 | No |
| 36 | FHL2 |  |  | 3383 | 0.013 | 0.0052 | No |
| 37 | RBBP8 |  |  | 3390 | 0.013 | 0.0065 | No |
| 38 | PDLIM3 |  |  | 3503 | 0.011 | -0.0047 | No |
| 39 | MREG |  |  | 3513 | 0.011 | -0.0041 | No |
| 40 | FKBP5 |  |  | 3790 | 0.005 | -0.0351 | No |
| 41 | IGF1R |  |  | 3930 | 0.002 | -0.0508 | No |
| 42 | FDFT1 |  |  | 3979 | 0.001 | -0.0562 | No |
| 43 | FASN |  |  | 4033 | 0.000 | -0.0622 | No |
| 44 | SNX24 |  |  | 4070 | -0.001 | -0.0663 | No |
| 45 | LRIG1 |  |  | 4165 | -0.002 | -0.0767 | No |
| 46 | XBP1 |  |  | 4331 | -0.006 | -0.0949 | No |
| 47 | TIAM1 |  |  | 4472 | -0.008 | -0.1097 | No |
| 48 | FOS |  |  | 4536 | -0.010 | -0.1155 | No |
| 49 | ISG20L2 |  |  | 4573 | -0.010 | -0.1181 | No |
| 50 | SIAH2 |  |  | 4677 | -0.012 | -0.1281 | No |
| 51 | FLNB |  |  | 4682 | -0.012 | -0.1267 | No |
| 52 | FKBP4 |  |  | 4706 | -0.013 | -0.1274 | No |
| 53 | AFF1 |  |  | 4800 | -0.014 | -0.1359 | No |
| 54 | CANT1 |  |  | 4955 | -0.017 | -0.1510 | No |
| 55 | SLC7A5 |  |  | 4959 | -0.017 | -0.1487 | No |
| 56 | RETREG1 |  |  | 5132 | -0.021 | -0.1653 | No |
| 57 | REEP1 |  |  | 5289 | -0.024 | -0.1796 | No |
| 58 | TIPARP |  |  | 5417 | -0.027 | -0.1901 | No |
| 59 | STC2 |  |  | 5456 | -0.028 | -0.1903 | No |
| 60 | MYBBP1A |  |  | 5465 | -0.028 | -0.1870 | No |
| 61 | INPP5F |  |  | 5488 | -0.028 | -0.1853 | No |
| 62 | AR |  |  | 5543 | -0.029 | -0.1870 | No |
| 63 | NXT1 |  |  | 5675 | -0.032 | -0.1972 | No |
| 64 | NRIP1 |  |  | 5868 | -0.035 | -0.2139 | No |
| 65 | B4GALT1 |  |  | 5870 | -0.036 | -0.2086 | No |
| 66 | NADSYN1 |  |  | 5963 | -0.037 | -0.2136 | No |
| 67 | MED13L |  |  | 5979 | -0.038 | -0.2096 | No |
| 68 | ABCA3 |  |  | 6089 | -0.040 | -0.2161 | No |
| 69 | CXCL12 |  |  | 6173 | -0.042 | -0.2192 | No |
| 70 | FARP1 |  |  | 6319 | -0.045 | -0.2290 | No |
| 71 | UGCG |  |  | 6331 | -0.045 | -0.2234 | No |
| 72 | MED24 |  |  | 6379 | -0.047 | -0.2218 | No |
| 73 | ADD3 |  |  | 6818 | -0.059 | -0.2631 | No |
| 74 | GJA1 |  |  | 6876 | -0.061 | -0.2604 | No |
| 75 | OLFM1 |  |  | 6897 | -0.062 | -0.2533 | No |
| 76 | ELOVL5 |  |  | 7151 | -0.069 | -0.2719 | No |
| 77 | SLC26A2 |  |  | 7256 | -0.073 | -0.2728 | No |
| 78 | HES1 |  |  | 7301 | -0.074 | -0.2665 | No |
| 79 | ABHD2 |  |  | 7643 | -0.088 | -0.2924 | Yes |
| 80 | WFS1 |  |  | 7649 | -0.088 | -0.2796 | Yes |
| 81 | SYNGR1 |  |  | 7720 | -0.092 | -0.2737 | Yes |
| 82 | DHCR7 |  |  | 7806 | -0.096 | -0.2689 | Yes |
| 83 | SLC39A6 |  |  | 7895 | -0.101 | -0.2637 | Yes |
| 84 | CA12 |  |  | 7910 | -0.102 | -0.2498 | Yes |
| 85 | PODXL |  |  | 8007 | -0.108 | -0.2446 | Yes |
| 86 | CELSR1 |  |  | 8013 | -0.108 | -0.2288 | Yes |
| 87 | AMFR |  |  | 8037 | -0.109 | -0.2148 | Yes |
| 88 | CHPT1 |  |  | 8084 | -0.113 | -0.2030 | Yes |
| 89 | AKAP1 |  |  | 8127 | -0.116 | -0.1902 | Yes |
| 90 | ENDOD1 |  |  | 8196 | -0.121 | -0.1796 | Yes |
| 91 | SEMA3B |  |  | 8382 | -0.144 | -0.1790 | Yes |
| 92 | GLA |  |  | 8383 | -0.144 | -0.1571 | Yes |
| 93 | IL6ST |  |  | 8394 | -0.146 | -0.1361 | Yes |
| 94 | TPBG |  |  | 8401 | -0.146 | -0.1146 | Yes |
| 95 | SLC16A1 |  |  | 8415 | -0.148 | -0.0936 | Yes |
| 96 | MYOF |  |  | 8488 | -0.159 | -0.0778 | Yes |
| 97 | SLC2A1 |  |  | 8552 | -0.173 | -0.0587 | Yes |
| 98 | PRSS23 |  |  | 8688 | -0.233 | -0.0389 | Yes |
| 99 | CD44 |  |  | 8778 | -0.337 | 0.0021 | Yes |
Table: GSEA details [plain text format]

  

Fig 2: HALLMARK\_ESTROGEN\_RESPONSE\_EARLY: Random ES distribution      
 Gene set null distribution of ES for **HALLMARK\_ESTROGEN\_RESPONSE\_EARLY**
